# BMC *Bioinformatics* reviewer acknowledgement 2015

**DOI:** 10.1186/s12859-016-0936-6

**Published:** 2016-02-09

**Authors:** Dirk Krüger

**Affiliations:** BioMed Central, Floor 6, 236 Gray’s Inn Road, London, WC1X 8HB UK

## Abstract

The editors of BMC Bioinformatics would like to thank all our reviewers who have contributed to the journal in Volume 16 (2015).

**Suyu Mei**

China

**Alexey Goltsov**

UK

**Andrzej Kierzek**

UK

**Adam P Levine**

UK

**Alan Wee-Chung Liew**

Australia

**Andrew Wood**

UK

**Andrej-Nikolai Spiess**

Germany

**Andrej Spiess**

Germany

**Aalt Van Dijk**

Netherlands

**Aaron Shafer**

Sweden

**Andreas Bender**

UK

**Alexej Abyzov**

USA

**Andrew C Doxey**

Canada

**Ariel Chernomoretz**

Argentina

**Alice Cleynen**

USA

**Andrew Cohen**

USA

**Anthony Cox**

UK

**Adrian Altenhoff**

Switzerland

**Alexandre Dufour**

France

**Ana Rita Grosso**

Portugal

**Abhinav Grover**

India

**Alexander Gusev**

USA

**Andrew Hart**

Chile

**Kamesh Arumugam**

USA

**Alla Karnovsky**

USA

**Andriy Kryshtafovych**

USA

**Alain Hauser**

Switzerland

**Alba Cristina De Melo**

Brazil

**Aleksey Zimin**

USA

**Alexander Dobrovic**

Australia

**Alex Valm**

USA

**Alexander Fletcher**

UK

**Alexander Wolff**

Germany

**Alexandre Bureau**

Canada

**Ali Anaissi**

Australia

**Sharma Alok**

Australia

**Stephen Altschul**

USA

**Alun Thomas**

USA

**Amarda Shehu**

USA

**Fernando Amat**

USA

**Anthony Mathelier**

Canada

**Amjad Ali**

Pakistan

**Ana Pavel**

USA

**Ananda Roy**

USA

**Ananth Kalyanaraman**

USA

**Anders Berglund**

USA

**Andigoni Malousi**

Greece

**András Aszódi**

Austria

**Andras Fiser**

USA

**Andrea Rau**

France

**Andreas Heger**

UK

**Andreas Keller**

Germany

**Andreas Prlic**

USA

**Andrei Lupas**

Germany

**E. Andres Houseman**

USA

**Andrew Dowsey**

UK

**Andrew Jaffe**

USA

**Andrew Allen**

USA

**Andrew Thomas**

Brazil

**Anoop Mayampurath**

USA

**Anna Goldenberg**

Canada

**Anna Ritz**

USA

**Anne Biton**

USA

**Anton Camacho**

UK

**Antonio Jose Jimeno Yepes**

Australia

**Ao Li**

China

**Alexandre Francisco**

Portugal

**Andrey Ptitsyn**

Qatar

**Alejandro Nato**

USA

**Lucia Chemes**

Argentina

**Ari Loytynoja**

Finland

**Arkadiusz Gertych**

USA

**Arndt Von Haeseler**

Austria

**Arne Elofsson**

Dominica

**Lars Arvestad**

Sweden

**Andreas Schlueter**

Germany

**Alexander Sczyrba**

Germany

**Mohammad Khan**

Malaysia

**Sourav S Bhowmick**

Singapore

**Alan Talevi**

Argentina

**Georgios Athanasiadis**

Denmark

**Hannah Nicholas**

Australia

**Merridee Wouters**

Australia

**Lachlan Coin**

Australia

**Scott Ritchie**

Australia

**Shu-Kay Angus Ng**

Australia

**Vanessa Teague**

Australia

**Matthew Ritchie**

Australia

**Xiaohui Tao**

Australia

**Andrew Collins**

Australia

**Louis Sherman**

Australia

**David Duffy**

Australia

**David Balding**

Australia

**Konsta Duesing**

Australia

**Ritaban Dutta**

Australia

**Yadav Sapkota**

Australia

**Gábor Mészáros**

Austria

**Florian Pauler**

Austria

**Rainer Schuhmacher**

Austria

**Gregory Ewing**

Austria

**Christian Partl**

Austria

**Avinash K Shanmugam**

USA

**Benjamin Chain**

UK

**Benjamin Linard**

UK

**Bas Van Breukelen**

Netherlands

**Rolf Backofen**

Germany

**Bairong Shen**

China

**Sandro Banfi**

Italy

**Zhirong Bao**

USA

**Basir Shariat**

USA

**Daniel Baum**

Germany

**Babajan Banaganapalli**

Saudi Arabia

**Robert Beiko**

Canada

**Jan De Neve**

Belgium

**Yvan Saeys**

Belgium

**Dieter Deforce**

Belgium

**Karoline Faust**

Belgium

**Gipsi Lima Mendez**

Belgium

**Benedicte Elena-Herrmann**

France

**Benoit Ballester**

France

**Nir Ben Tal**

Israel

**Bernd Fischer**

Germany

**Denis Bertrand**

Singapore

**Bharath Srinivasan**

USA

**Binghuang Cai**

USA

**Velumani Bhuvaneswari**

India

**Ali Bilgin**

USA

**Binay Panda**

India

**Bingqiang Liu**

Canada

**Bingshan Li**

USA

**Vijay Garg**

India

**Monika Michalovova**

USA

**Bernard Pope**

Australia

**Burkhard Morgenstern**

Germany

**Ornulf Borgan**

Norway

**Anne-Laure Boulesteix**

Germany

**Babak Alipanahi**

Canada

**Bo Peng**

USA

**Yu Xia**

Canada

**Douglas Pires**

Brazil

**Hernandes Carvalho**

Brazil

**Benilton Carvalho**

Brazil

**Raydonal Ospina**

Brazil

**Daniel Oliveira**

Brazil

**Brett Lidbury**

Australia

**Brian Connolly**

USA

**Fiona Brinkman**

Canada

**Bruno Nevado**

UK

**Brian Tjaden**

USA

**Veselka Boeva**

Bulgaria

**Donald H Burke**

USA

**Carsten Kemena**

Germany

**Can Kesmir**

Netherlands

**Chanchal Mitra**

India

**Chunyu Jin**

USA

**Bhagwati Gupta**

Canada

**Earl Brown**

Canada

**Christian Frech**

Canada

**Herbert H Tsang**

Canada

**Dionne Aleman**

Canada

**Marek Laskowski**

Canada

**Jennifer Mitchell**

Canada

**Hagit Shatkay**

Canada

**Shaun Jackman**

Canada

**Alan Moses**

Canada

**Donovan Parks**

Canada

**Xuhua Xia**

Canada

**Carl Kingsford**

USA

**Carlo Manzo**

Spain

**Carlo Baldassi**

Italy

**Carolina Wahlby**

USA

**Rita Casadio**

Italy

**Chris Benner**

USA

**Christian Blouin**

Canada

**Carlos Castro**

Spain

**Celia Greenwood**

Canada

**Ching-Fen Jiang**

Taiwan

**Brad Chapman**

USA

**Charlotte Soneson**

Switzerland

**Chava Kimchi-Sarfaty**

USA

**Jun Chen**

USA

**Chen Kaesar**

Israel

**Cheng Chen**

USA

**Jianlin Cheng**

USA

**Yizong Cheng**

USA

**Chiara Romualdi**

Italy

**Chih-Hsuan Wei**

USA

**Chih Lee**

USA

**Rayan Chikhi**

France

**Jin Gu**

China

**Zhi-Ping Liu**

China

**Yinglei Song**

China

**Xingming Zhao**

China

**Ming Chen**

China

**Huaqiang Du**

China

**Zhi Lu**

China

**Yc Wang**

China

**Xuan Xiao**

China

**Zu-Guo Yu**

China

**Min Li**

China

**Yaning Yang**

China

**Wei Chen**

China

**Quan Zou**

China

**Yan Fu**

China

**Xuehai Hu**

China

**Yihui Luan**

China

**Lei Qu**

China

**Hong-Bin Shen**

China

**Yi Liu**

China

**Shaowu Zhang**

China

**Zhiwei Cao**

China

**Rui Jiang**

China

**Yanghua Xiao**

China

**Jianhua Xu**

China

**Yu Xue**

China

**Simon Rayner**

China

**Wei Jiang**

China

**Fei Liu**

China

**Ching Wan Lam**

Hong Kong

**Chloé-Agathe Azencott**

France

**Christian Paroissin**

France

**Christian Remmele**

Germany

**Christian Schlötterer**

Austria

**Christine Nardini**

Italy

**Christine Staiger**

Netherlands

**Ambroise Christophe**

France

**Christophe Beroud**

France

**Christopher Funk**

USA

**Chuck Cannon**

USA

**James Cimino**

USA

**Cinzia Pizzi**

Italy

**Cinzia Viroli**

Italy

**Charles Kervrann**

France

**Caleb Stein**

USA

**Claudio Donati**

Italy

**Christina Leslie**

USA

**Che-Lun Hung**

Taiwan

**Cristina Marino-Buslje**

Argentina

**Carlos Silla**

Brazil

**Hanbo Chen**

USA

**Colin Clarke**

Ireland

**Colin Smith**

Germany

**Matteo Comin**

Italy

**Oliver Philipp**

Germany

**Cheolwoo Park**

USA

**Cedric Saule**

Germany

**Casey Greene**

USA

**C. Titus Brown**

USA

**Ching-Ti Liu**

USA

**Qing-Hua Cui**

China

**Fabio Cunial**

Germany

**Chris Upton**

Canada

**Ching-Wei Wang**

Taiwan

**Cyril Grouin**

France

**Chenggang Yu**

USA

**Jan Brezovsky**

Czech Republic

**Jiri Klema**

Czech Republic

**Diana Hendrickx**

Netherlands

**Donovan Parks**

Australia

**Thomas Dandekar**

Germany

**Daniel Aguilar**

USA

**Daniel Sage**

Switzerland

**Daniel Falush**

UK

**David Price**

USA

**David Ardell**

USA

**David Blair**

USA

**David La**

USA

**David Hall**

USA

**David Meckes**

USA

**David A Eberhard**

USA

**Davide Risso**

USA

**Danny Barash**

Israel

**Dukka Kc**

USA

**Debasisa Mohanty**

India

**Deepak Ayyala**

USA

**Deepak Singla**

USA

**Denis Bauer**

Australia

**Huangdi Yi**

China

**Jonas Andreas Sibbesen**

Denmark

**Dennis Hazelett**

USA

**Dennis Welker**

USA

**Des Higgins**

Ireland

**Deyvid Amgarten**

Brazil

**Domenico L Gatti**

USA

**Damyanthi Herath**

Australia

**Dragos Horvath**

France

**Diana Bahia**

Brazil

**Diana Sima**

Belgium

**Dick De Ridder**

Netherlands

**Susan Ghiassian**

USA

**Dinesh Kumar**

USA

**Dirk Valkenborg**

Belgium

**Daofeng Li**

USA

**Daniel Machado**

Portugal

**Dominik Heider**

Germany

**Dominik Schrempf**

Austria

**Nadezhda Doncheva**

Germany

**Dong Si**

USA

**Dong Wang**

USA

**Deguang Kong**

USA

**Subramanian Krishnakumar**

India

**Dean Rowe-Magnus**

USA

**Dario Strbenac**

Australia

**Deqiang Sun**

USA

**Duccio Fanelli**

Italy

**Murat Dundar**

USA

**Micah Dunthorn**

Germany

**Dannie Durand**

USA

**Derrick Wood**

USA

**Di Wu**

USA

**Ingo Ebersberger**

Germany

**Edyta Koscianska**

Poland

**Eldon Emberly**

Canada

**Can Yang**

USA

**Ralf Eggeling**

Germany

**Etienne Kornobis**

Germany

**Eleni Giannoulatou**

UK

**Eduardo Andrés-León**

Spain

**Emily Prud'Hommeaux**

USA

**Emily Chia-Yu Su**

Taiwan

**Ellis Patrick**

USA

**Erica Marie Hartmann**

USA

**Erich Baker**

USA

**Eric Wait**

USA

**Erliang Zeng**

USA

**Esmaeil Ebrahimie**

Australia

**Eric Vallender**

USA

**Eytan Domany**

Israel

**Fuyong Xing**

USA

**Fabian Sievers**

Ireland

**Falk Hildebrand**

Germany

**Falk Schreiber**

Australia

**Ruzong Fan**

USA

**Mario Fares**

Ireland

**Alexander Favorov**

Russia

**Francesca Cordero**

Italy

**Francisco Couto**

Portugal

**Feifan Liu**

USA

**Francisco Enguita**

Portugal

**Ferhat Ay**

USA

**Filippo Utro**

Switzerland

**Pekka Marttinen**

Finland

**Petri Toronen**

Finland

**Fiona McCarthy**

USA

**Federico Giorgi**

USA

**Folker Meyer**

USA

**Dimitrios Fotiadis**

Greece

**Francesco Pappalardo**

Italy

**Guy Perriere**

France

**Yann Ponty**

France

**Eva Dhondt**

France

**Julien Gobeill**

France

**Benjamin Roche**

France

**Guillaume Achaz**

France

**Arthur Tenenhaus**

France

**Daniel Jost**

France

**Pierre Peterlongo**

France

**Alexandre De Brevern**

France

**Bruno Toupance**

France

**Thomas Boudier**

France

**Laurent Brehelin**

France

**Mathieu Gautier**

France

**Claudine Medigue**

France

**Caroline Berard**

France

**Francesca Martella**

Italy

**Tzong-Yi Lee**

Taiwan

**Francisco De La Vega**

USA

**Franck Picard**

France

**Fred Hamprecht**

Germany

**Saskia Freytag**

Australia

**Holger Fröhlich**

Germany

**Feixiong Cheng**

USA

**Gibran Hemani**

UK

**Gabriel Cristóbal**

Spain

**Gabriel Renaud**

Germany

**Julien Gagneur**

Germany

**Ganesh Sahoo**

India

**Vincent Gardeux**

Switzerland

**Gerard Cagney**

Ireland

**Michaela Bayerlova**

Germany

**Ulrich Stelzl**

Germany

**Manuel Holtgrewe**

Germany

**André Scherag**

Germany

**Jochen Blom**

Germany

**Jaime Huerta-Cepas**

Germany

**Bryan Downie**

Germany

**Marcel Martin**

Germany

**Harald Binder**

Germany

**Wolfgang Förstner**

Germany

**Stefan Paulus**

Germany

**Axel Benner**

Germany

**Julien Dutheil**

Germany

**Friedrich Foerster**

Germany

**Frank Kramer**

Germany

**Astrid Wachter**

Germany

**Marcus Dittrich**

Germany

**Marcus Lechner**

Germany

**Mariana Neves**

Germany

**Ezgi Karaca**

Germany

**Benedikt Brors**

Germany

**Matthias Ganzinger**

Germany

**Jerzy Dyczkowski**

Germany

**Kaustubh Patil**

Germany

**Ursula Kummer**

Germany

**Johannes Köster**

Germany

**Michael Seifert**

Germany

**Graham Hatfull**

Brazil

**Greg Finak**

USA

**Giorgio Bertorelle**

Italy

**Ghislain Bidaut**

France

**Gianfranco Politano**

Italy

**Georgi Marinov**

USA

**George Michailidis**

USA

**Gokmen Zararsiz**

Turkey

**Mehmet Gonen**

USA

**Gonzalo Ruz**

Chile

**Georgios Tsaousis**

Greece

**Margarita Theodoropoulou**

Greece

**Gordon Robertson**

Canada

**Michael Gromiha**

India

**Gustavo Rosania**

USA

**Thierry Zozio**

France

**Guillemette Marot**

France

**Gur Yaari**

Israel

**Gyorgy Abrusan**

Hungary

**Yuanfang Guan**

USA

**Holly Bik**

UK

**Henry Wood**

UK

**Hien Nguyen**

Australia

**Hae Uh**

Netherlands

**Ran Libeskind-Hadas**

USA

**Chen Haifeng**

China

**Hailin Chen**

USA

**Haiquan Li**

USA

**Hajk-Georg Drost**

Denmark

**Thierry Hamon**

France

**Hanchuan Peng**

USA

**Hane Lee**

USA

**Jikai Lei**

USA

**Hannah Christensen**

UK

**Hans Kestler**

Germany

**Hao Zheng**

USA

**M Hasinur Rahaman Khan**

Bangladesh

**Haixu Tang**

USA

**Bernhard Haubold**

Germany

**Hao Chen**

Hong Kong

**Huaichun Wang**

Canada

**Heba Saadeh**

UK

**Heewook Lee**

USA

**Heike Siebert**

Germany

**Heiner Klingenberg**

Germany

**Heng Wang**

USA

**Heping Zhang**

USA

**Hesam Montazeri**

Switzerland

**Itaru Hirai**

Japan

**Hyun Jung Park**

USA

**Hiqmet Kamberaj Macedonia**

The Former Yugoslav Republic Of

**Hailong Zhu**

China

**Hidetoshi Matsui**

Japan

**Hongmin Cai**

China

**Haimao Zhan**

USA

**Xiaowen Chu**

Hong Kong

**Xiaodan Fan**

Hong Kong

**Hongyu Zhao**

USA

**Roman Hornung**

Germany

**Lin Hou**

USA

**Houtan Noushmehr**

Brazil

**Hai Su**

USA

**Xia Hu**

USA

**Hu Qian-Nan**

China

**Hui Huang**

USA

**Yuan Huang**

USA

**Hunter Moseley**

USA

**Hongyan Xu**

USA

**Hamid Younesy**

Canada

**Henry Zhang**

USA

**Hongyi Zhou**

USA

**Iain Manfield**

UK

**Ihor Smal**

Netherlands

**Thomas Simpson**

UK

**Iddo Friedberg**

USA

**Ivan Dotu**

USA

**Ilya Serebriiskii**

USA

**Ilan Samish**

Isreal

**Ioannis Iliopoulos**

Greece

**Imhoi Koo**

USA

**Ina Koch**

Germany

**Narayanaswamy Srinivasan**

India

**Hamsa Priya Mohana Sundaram**

India

**Gajendra Raghava**

India

**Achuthsankar S Nair**

India

**Rameshkumar Krishnamoorthy**

India

**B. Jayaram**

India

**Balraj Mittal**

India

**Inke König**

Germany

**Inken Wohlers**

Germany

**Ion Mandoiu**

USA

**Cathal Seoighe**

Ireland

**Dietrich Rebholz-Schuhmann**

Ireland

**Irene Tischer**

Colombia

**Isaam Saeed**

Australia

**Tal Pupko**

Israel

**Firas Swidan**

Israel

**Sol Efroni**

Israel

**Eyal Privman**

Israel

**Tamir Tuller**

Israel

**Giorgio Valentini**

Italy

**Luisa Di Paola**

Italy

**Flavia Guzzo**

Italy

**Silvio Tosatto**

Italy

**Cristian Del Fabbro**

Italy

**Piero Fariselli**

Italy

**Pier Luigi Martelli**

Italy

**Pietro Di Lena**

Italy

**Andrea Calabria**

Italy

**Pasquapina Ciarmela**

Italy

**Serafina Massari**

Italy

**Kimihito Ito**

Japan

**Ivan Kulakovskiy**

Russia

**Ivan Limongelli**

Italy

**Ivan Merelli**

Italy

**Jörg Ackermann**

Germany

**Jiyuan An**

USA

**Pjotr Prins**

Netherlands

**Jorge Duitama**

Colombia

**Yu-Chieh Liao**

Taiwan

**Nivedita Deo**

USA

**James Costello**

USA

**James Lara**

USA

**Jian Zhang**

USA

**Jamie Sherman**

Canada

**Jan Fostier**

Belgium

**Jan Hasenauer**

Germany

**Naoko Takezaki**

Japan

**Fumihiko Takeuchi**

Japan

**Masanori Arita**

Japan

**Wataru Nemoto**

Japan

**Yoshimasa Tsuruoka**

Japan

**Nozomi Nagano**

Japan

**Timo Lassmann**

Japan

**Jun Zhang**

Japan

**Michiaki Hamada**

Japan

**Yang Yang**

Japan

**Tadashi Ando**

Japan

**Satya Arjunan**

Japan

**Jared Simpson**

Canada

**Jaroslaw Zola**

USA

**Javed Agrewala**

India

**Jayajit Das**

USA

**Jared Burks**

USA

**João André Carriço**

Portugal

**Jair Cervantes**

Mexico

**Jason Kahn**

USA

**Jean Yang**

Australia

**Jean-Christophe Lambry**

France

**Jean-Jacques Daudin**

France

**Robert Jernigan**

USA

**Jérôme Ambroise**

Belgium

**Jianfeng Pei**

China

**Jianjiong Gao**

USA

**John Hartman**

USA

**Jiangning Song**

Australia

**Yu Jiang**

USA

**Jiehuan Sun**

USA

**Jieyue Li**

USA

**Jinbo Xu**

USA

**Jin Chu Wu**

USA

**Jing Tang**

Finland

**Jing-Hao Xue**

UK

**Victor Jin**

USA

**Jin Yu Li**

USA

**Jonas Korlach**

USA

**Jie Liang**

USA

**John-Marc Chandonia**

USA

**Jarek Meller**

USA

**John Murray**

USA

**Juanma Vaquerizas**

Germany

**Joan Maynou**

Spain

**Joel Tellinghuisen**

USA

**Joeri Ruyssinck**

Belgium

**Johannes Droege**

Germany

**Johannes Waage**

Denmark

**Jonathan Keith**

USA

**Jos Boekhorst**

Netherlands

**Joshua Payne**

Switzerland

**Jean Peccoud**

USA

**Jianhua Ruan**

USA

**Junhee Seok**

South Korea

**Jiansen Jiang**

USA

**José Patané**

Brazil

**Florian Jug**

Germany

**Juilee Thakar**

USA

**Julia Perera Bel**

Germany

**Juliana Bernardes**

France

**Julio Banga**

USA

**Suckjoon Jun**

USA

**Justin Zook**

USA

**James Taylor**

USA

**Jianxin Wang**

China

**Jingyuan Song**

China

**Janos Zempleni**

USA

**Kim-Anh Lê Cao**

Australia

**Kristina Hettne**

Netherlands

**Koji Kadota**

Japan

**Karin Verspoor**

Australia

**Karina S. Machado**

Brazil

**Karsten Hiller**

Luxembourg

**Katerina Kechris**

USA

**Kazutaka Katoh**

Japan

**Kent Riemondy**

USA

**Karen Ross**

USA

**Kevin Yip**

Hong Kong

**Glass K**

USA

**Kin Fai Au**

USA

**Kirk Roberts**

USA

**Kishore Mosaliganti**

USA

**Kevin Keegan**

USA

**Pavel Skums**

USA

**Klaus Jung**

Germany

**Marius Kloft**

Germany

**Kailin Tang**

China

**Karina Mayorga**

Mexico

**Tamas Korcsmaros**

Hungary

**Sukjoon Yoon**

South Korea

**Sun Shim Choi**

South Korea

**Buhm Han**

South Korea

**Jaehee Kim**

South Korea

**Wonkuk Kim**

South Korea

**Sergey Koren**

USA

**Kostas Tsirigos**

Sweden

**Khalid Raza**

India

**Srikrishna Subramanian**

India

**Kristin Ayers**

UK

**Kristof Engelen**

Belgium

**Sergey Ovchinnikov**

USA

**Kristoffer Sahlin**

Sweden

**Konstantinos Spiliopoulos**

USA

**Kalliopi Tsafou**

USA

**Kentaro Tomii**

Japan

**Stefan Kurtz**

Germany

**Anthony Kusalik**

Canada

**Kai Wang**

USA

**Ken Witwer**

USA

**Yung-Keun Kwon**

South Africa

**Kristine Wylie**

USA

**Kazunori Yamada**

Japan

**Lan Du**

Australia

**Lars-Gustav Snipen**

Norway

**Lars Bongo**

Norway

**Jessica Larson**

USA

**Laura Saba**

USA

**Laurent Jacob**

France

**Mario Lauria**

Italy

**Layla Oesper**

USA

**Leandro Guerrero**

South Africa

**Robert Hoehndorf**

UK

**Lee Kamentsky**

USA

**Seunggeun Lee**

USA

**Lei Song**

USA

**Leighton Pritchard**

UK

**Matej Lexa**

Czech Republic

**Luís F De Figueiredo**

UK

**Lana Garmire**

USA

**Li Chen**

USA

**Li Beißbarth**

Germany

**Li Wang**

USA

**Xing Li**

USA

**Chun Liang**

USA

**Guangdi Li**

China

**Lili Wang**

Canada

**Lin Xu**

USA

**Xiang Li**

USA

**Yushi Liu**

USA

**Yazhi Liu**

China

**Liyang Diao**

USA

**Ying Li**

China

**Kenli Li**

China

**Long Qu**

USA

**Loredana Martignetti**

France

**Lorenzo Tattini**

Italy

**Luca Pinello**

USA

**Lynn Schriml**

USA

**Li Song**

USA

**Liang Hu Qu**

China

**Lu Huang**

USA

**Lucas Carey**

Spain

**Luca Toldo**

Germany

**Luciano Cascione**

Switzerland

**Luis Sanchez-Pulido**

UK

**Luke Czapla**

USA

**Lun-Ching Chang**

USA

**Jiawei Luo**

China

**Lei Xie**

USA

**Lynn Lin**

USA

**Minh Duc Cao**

Australia

**Martijn Huynen**

Netherlands

**Marek Kowal**

Poland

**Miron Kursa**

Poland

**Markus Meringer**

Germany

**Mark Wass**

UK

**Mark Ragan**

Australia

**Mar Rodriguez Girondo**

USA

**Mathieu Rouard**

Italy

**Monica Santamaria**

Italy

**Marc Santolini**

USA

**Marco Scutari**

UK

**Nadia El-Mabrouk**

Canada

**Kathy Macropol**

USA

**Mahreen Arooj**

Australia

**Wolfgang Gerlach**

USA

**Majid Kazemian**

USA

**George Makhatadze**

USA

**Manabu Torii**

USA

**Manish Kumar**

India

**Giovanni Manzini**

Italy

**Marcella Attimonelli**

Italy

**Blanca Caminero**

Spain

**Marie-Paule Lefranc**

France

**Mark D. Robinson**

Switzerland

**Mark Van De Wiel**

Netherlands

**Martin Hofmann-Apitius**

Germany

**Martin Maska**

Czech Republic

**Martin Weigt**

France

**Joaquim Martins Jr**

Brazil

**Martin Mascher**

Germany

**Masaaki Kotera**

Japan

**Matteo Benelli**

Italy

**Matteo Figliuzzi**

France

**Matthew Bennett**

USA

**Matthew Macmanes**

USA

**Matthew McCall**

USA

**Matthias Wolf**

Germany

**Louxin Zhang**

Singapore

**Maxim Barenboim**

Germany

**Johanna Mazur**

Germany

**Manoj Bhasin**

USA

**Min Chen**

USA

**Maria Chikina**

USA

**Paul McMurdie**

USA

**Matthias Döring**

Germany

**Mark Ebbert**

USA

**Megan Orr**

USA

**Meik Kunz**

Germany

**Menachem Fromer**

USA

**Mengjie Chen**

USA

**Enrique Hernandez-Lemus**

Mexico

**Rafael Villalobos**

Mexico

**Michelle Giglio**

USA

**Mohammad Ganjtabesh**

Iran

**Melissa Gymrek**

USA

**Michael Herman**

USA

**Micha Bayer**

UK

**Michael Götze**

Germany

**Michael Hoopmann**

USA

**Michal Okoniewski**

Switzerland

**Michal Brylinski**

USA

**Michelangelo Ceci**

Italy

**Mikael Salson**

France

**Mikhail Spivakov**

UK

**Mile Sikic**

Croatia

**Ming Hu**

USA

**Minh Nguyen**

Singapore

**Ramanathan Sowdhamini**

India

**Mira Han**

USA

**Julie Mitchell**

USA

**Matloob Khushi**

Australia

**Mahbubul Majumder**

USA

**Ram Podicheti**

USA

**Mohamed Elati**

France

**Monica Chagoyen**

Spain

**Morihiro Hayashida**

Japan

**Marco Punta**

UK

**Reaz Uddin**

Pakistan

**Madis Rumming**

Germany

**Moisés Santillan**

Mexico

**Marcel H. Schulz**

Germany

**Margaret Taub**

USA

**Mu Gao**

USA

**T. Murali**

USA

**Mark Winter**

USA

**Maria-Eirini Pandelia**

USA

**Nadim Ajami**

USA

**Nima Aghaeepour**

USA

**Ali Najafi**

Iran

**Nam Nguyen**

USA

**Nan Zhao**

USA

**Nanjiang Shu**

Sweden

**Natali Gulbahce**

USA

**Nathalie Villa-Vialaneix**

France

**Nathaniel Snyder**

USA

**Saket Navlakha**

USA

**Neo Christopher Chung**

USA

**Neil Mac Parthaláin**

UK

**Andrew Wong**

USA

**Nestoras Karathanasis**

Greece

**Gert Vriend**

Netherlands

**Peter Horvatovich**

Netherlands

**K. Anton Feenstra**

Netherlands

**Casper Albers**

Netherlands

**Hinda Haned**

Netherlands

**David Welch New**

Zealand

**Nicola Segata**

Italy

**Nicolas Heck**

France

**Ning Chen**

China

**Nianjun Teng**

China

**Ning Leng**

USA

**Nan Li**

USA

**Nan Lin**

USA

**Daniel Noguera**

USA

**Finn Drabløs**

Norway

**Nico Pfeifer**

Germany

**Nima Pouladi**

USA

**Nurcan Tuncbag**

USA

**Grégory Nuel**

France

**Ruth Nussinov**

USA

**Osamu Gotoh**

Japan

**Olga Vitek**

USA

**Catalina Tudor**

USA

**Jonathon O'Brien**

USA

**David Ochoa**

UK

**Michael Ochs**

USA

**Olivier Cinquin**

USA

**Olivier Gevaert**

USA

**Kazuma Okada**

Japan

**Olaf Bininda-Emonds**

Germany

**Oliver Schilling**

Germany

**Omer An**

UK

**Brian Ondov**

USA

**Ong Mei-Lyn**

Singapore

**Oswaldo Trelles**

Spain

**Piotr Makowski**

Poland

**Pablo Minguez**

Germany

**Paul Aiyetan**

USA

**Pal Sætrom**

Norway

**Palok Aich**

India

**Bogdan Pasaniuc**

USA

**Andrea Passerini**

Italy

**Patricio Oyarzun**

Chile

**Patthy Laszlo**

Hungary

**Paul Boutros**

Canada

**Paula Veríssimo**

Portugal

**Florencio Pazos**

Spain

**Patrick Chain**

USA

**Piotr Cieplak**

USA

**Marcin Skwark**

Finland

**Shyamal Peddada**

USA

**Pedro Ballester**

France

**Peng Qiu**

USA

**Peter Bajcsy**

USA

**Peter White**

USA

**Petra Knaup**

Germany

**Phil Lee**

USA

**Philippe Gambette**

France

**Barry Smith**

USA

**Pietro Franceschi**

Italy

**Pingzhao Hu**

USA

**Pingzhao Hu**

Canada

**Peng Jiang**

USA

**Paulo Pierry**

Brazil

**Ilona Wandzik**

Poland

**Henryk Rybinski**

Poland

**Sandor Pongor**

Italy

**Matthias Erwin Futschik**

Portugal

**Pratyaksha Wirapati**

Switzerland

**Predrag Radivojac**

USA

**Pedro Ribeiro**

Portugal

**Patrick Schloss**

USA

**P. Shanmugavadivu**

India

**Pierre Zweigenbaum**

France

**Ping Zhang**

USA

**Qian Wang**

USA

**Qibin Zhang**

USA

**Michele Ceccarelli**

Qatar

**Qiangjun Zhou**

USA

**Ji Qi**

China

**Qing Zhao**

USA

**Jing Qin**

Hong Kong

**Li-Xuan Qin**

USA

**Qiongshi Lu**

USA

**Jing Qiu**

USA

**Xin Qi**

USA

**Yifei Qi**

USA

**Quan Chen**

USA

**Quang Le**

UK

**Ranjit Bahadur**

India

**Roman Koning**

Netherlands

**Robert Nowak**

Poland

**Ralf Schmid**

UK

**Rachel Schwartz**

USA

**Rainer Merkl**

Germany

**Rainer Spang**

Germany

**Rakesh Joshi**

India

**Ramakrishnan C,**

India

**Ramon Diaz-Uriarte**

Spain

**Franck Rapaport**

USA

**Tobias Rausch**

Germany

**Ravi Shankar**

India

**Robert Rebhun**

USA

**Ren-Hua Chung**

Taiwan

**Rebecca Hunt**

Germany

**Reinhard Guthke**

Germany

**Remo Sanges**

Italy

**Rudy Guerra**

USA

**Riccardo Bellazzi**

Italy

**Richard Boys**

UK

**Richard White**

USA

**Rongjian Li**

USA

**Robert Frost**

USA

**Rob Patro**

USA

**Robert Kofler**

Austria

**Robert Leaman**

USA

**Robert Edgar**

USA

**Robert Cooper**

USA

**Rodolfo Negri**

Italy

**Rodrigo Guarischi-Sousa**

Brazil

**Rohit Vashisht**

USA

**Roland Krause**

Luxembourg

**Ron Wehrens**

Netherlands

**Rachid Ounit**

USA

**Raman Parkesh**

India

**Raul Tonda**

Spain

**Rui Zhong**

USA

**Ruslan Kalendar**

Finland

**Ekaterina Chernyaeva**

Russia

**Rohit Bhargava**

USA

**Rong Xu**

USA

**Shu-Kay Ng**

Australia

**Sebastian Schmeier**

New Zealand

**Shailesh Tripathi**

UK

**Sabarinathan Radhakrishnan**

Denmark

**Mohd Saberi Mohamad**

Malaysia

**Saeed Salem**

USA

**Salvador Capella-Gutíerrez**

Spain

**David Sankoff**

Canada

**Santi Garcia-Vallvé**

Spain

**Sarah Cohen Boulakia**

France

**Sarah Hird**

USA

**Sara Mostafavi**

Canada

**Sarwar Kamal**

Bangladesh

**Vladimir Bajic**

Saudi Arabia

**Sam Scarpino**

USA

**Alejandro Schaffer**

USA

**Alexander Schliep**

USA

**Scott Doyle**

USA

**Sebastian Kaiser**

USA

**Sebastian Maurer-Stroh**

Singapore

**Selvaraj Samuel**

India

**Jose Seoane**

USA

**Miljana Tanic**

Serbia

**Sergio Marco Garrido**

France

**Andrew Severin**

USA

**Shubhra Ghosh Dastidar**

India

**Sarah Goodwin**

USA

**Yuanpu Xie**

USA

**Yuk Sham**

USA

**Shandar Ahmad**

Japan

**Steffen Heber**

USA

**Shishi Luo**

USA

**Shishir Gupta**

Germany

**Xingjie Shi**

China

**Sheng Li**

USA

**Shuai Huang**

USA

**Shuangge Ma**

USA

**Siamak Tafavogh**

USA

**Silke Sperling**

Germany

**Simon Andrews**

UK

**Simona E. Rombo**

Italy

**Simona Rombo**

Italy

**Andreas Wilm**

Singapore

**Weimiao Yu**

Singapore

**Min Wu**

Singapore

**Janos Kriston-Vizi**

Singapore

**Lit-Hsin Loo**

Singapore

**Erik Cambria**

Singapore

**Jung-Jae Kim**

Singapore

**Shailza Singh**

India

**Samad Jahandideh**

USA

**Sidharth Chopra**

India

**Sara Madeira**

Portugal

**Simone Marsili**

Spain

**Sumit Middha**

USA

**Stefano Monti**

USA

**Gordon Smyth**

Australia

**Sorin Draghici**

USA

**Johannes Soeding**

Germany

**Solon Pissis**

UK

**Song Li**

USA

**Salvador Capella-Gutiérrez**

Spain

**Ivan Junier**

Spain

**Samira Jaeger**

Spain

**Juan Jesus Perez**

Spain

**Jana Selent**

Spain

**Marc A. Marti-Renom**

Spain

**David Juan**

Spain

**Pilar Gonzalez-Gomez**

Spain

**María Mar Abad-Grau**

Spain

**Pablo Porras Millan**

Spain

**Paul Spellman**

USA

**Son Pham**

USA

**Saurabh Prasad**

USA

**Dwight Stambolian**

USA

**Stefan Schulz**

Austria

**Steffen Sass**

Germany

**Stephan Pabinger**

Austria

**Stephane Dray**

France

**Steven Rozen**

Singapore

**Stephen Salipante**

USA

**Vincent Carey**

USA

**Weiliang Qiu**

USA

**Subhajit Sengupta**

USA

**Sunghwan Kim**

South Korea

**Sukarna Barua**

Bangladesh

**Zhifu Sun**

USA

**Sutirtha Chakraborty**

USA

**Suvendra Kumar Ray**

India

**David Svoboda**

Czech Republic

**Shi Wang**

China

**Swapna Mahurkar Joshi**

USA

**Mikael Benson**

Sweden

**Sebastian Will**

Germany

**Frederic Guillaume**

Switzerland

**Hubert Rehrauer**

Switzerland

**Federico Santoni**

Switzerland

**Sylvain Trépout**

France

**Silke Szymczak**

Germany

**Bor-Sen Chen**

Taiwan

**Chung-Feng Liu**

Taiwan

**Jui-Hung Hung**

Taiwan

**Kazuhiro Takemoto**

Taiwan

**Akito Taneda**

Japan

**Xiaojia Tang**

Japan

**Michel Tchuenche**

USA

**Tatiana Tatusova**

Tanzania

**Taylor Wilcox**

USA

**Toby Hocking**

USA

**Tuan Pham**

Canada

**Ruoqing Zhu**

Japan

**Teng Zhang**

USA

**Herve Tettelin**

USA

**Sissades Tongsima**

USA

**Tomas Helikar**

Thailand

**Theo Niyonsenga**

USA

**Thien Ho**

Australia

**Krishnaraj Thirugnanasambantham**

USA

**Thomas Faraut**

India

**Thomas Lingner**

France

**Thomas Pfau**

Germany

**Thomas Walter**

Luxembourg

**Julie Thompson**

France

**Tianqi Liu**

France

**Tsung-I Lin**

USA

**Tim Beißbarth**

Taiwan

**German Tischler**

Germany

**Tiziana Margaria**

Germany

**Thomas James Hardcastle**

Germany

**Tao Liu**

UK

**Tobias Müller**

USA

**Tobias Ölschläger**

Germany

**Kiehl Thomas**

Denmark

**Torben Nielsen**

USA

**Torbjorn Rognes**

USA

**Torsten Waldminghaus**

Norway

**Thomas Pengo**

Germany

**Tim Reviewer**

USA

**Olivia Reviewer**

UK

**Travis Gagie**

UK

**Todd Treangen**

Finland

**William Trimble**

USA

**Thomas Rindflesch**

USA

**Yoshimasa Tsuruoka**

USA

**Tom Tullius**

UK

**Arzucan Ozgur**

USA

**Osman Ugur Sezerman**

Turkey

**Guray Kuzu**

Turkey

**Gabor Tusnady**

Turkey

**Tzachi Hagai**

Hungary

**Uchiyama Ikuo**

UK

**Graham Ritchie**

Japan

**Simon Rogers**

UK

**Neil David Rawlings**

UK

**Magdalena Zarowiecki**

UK

**Lachlan Coin**

UK

**Makoto Miwa**

UK

**Silvia Liverani**

UK

**Vincent Plagnol**

UK

**Marion Dawn Teare**

UK

**Roman Laskowski**

UK

**Andrea Sottoriva**

UK

**David Balding**

UK

**Nicholas Furnham**

UK

**Diego Oyarzun**

UK

**Rebecca Cordell**

UK

**Katie Lunnon**

UK

**Gemma Sharp**

UK

**Ramon Grima**

UK

**Andreas Dr&Auml;Ger**

UK

**Bhagwati Gupta**

USA

**Rasna Walia**

USA

**Min He**

USA

**Yaping Liu**

USA

**Chun-Hsi Huang**

USA

**Eckart Bindewald**

USA

**Nathan Clement**

USA

**Yongchao Liu**

USA

**Yufeng Wu**

USA

**Jaques Reifman**

USA

**Gary Chen**

USA

**Christopher Hill**

USA

**Connor Skennerton**

USA

**Kristin Bennett**

USA

**Alice Wattam**

USA

**Tandy Warnow**

USA

**Swati Biswas**

USA

**Chen Wang**

USA

**Peng Yu**

USA

**Zohreh Talebizadeh**

USA

**Oncel Tuzel**

USA

**Moitrayee Bhattacharyya**

USA

**Chi Zhang**

USA

**Grzegorz Boratyn**

USA

**Dima Kozakov**

USA

**Cesar Ramirez**

USA

**Yang Dai**

USA

**Richard Gronostajski**

USA

**Matthew Nicholson McCall**

USA

**Ka Yee Yeung**

USA

**Yuzhen Ye**

USA

**Brett McKinney**

USA

**Ann Oberg**

USA

**Ryan Layer**

USA

**Hongyi Xin**

USA

**Giulio Genovese**

USA

**Po-Ru Loh**

USA

**Robert Page**

USA

**Huy Dinh**

USA

**Patrick Flaherty**

USA

**Jeremy Goecks**

USA

**Patricia Chan**

USA

**William Nes**

USA

**Stan Pounds**

USA

**Keith Dunker**

USA

**Serdar Bozdag**

USA

**Sawsan Khuri**

USA

**Yoo-Ah Kim**

USA

**Ting Chen**

USA

**Siddhartha Jonnalagadda**

USA

**Cecilia Arighi**

USA

**Hagit Shatkay**

USA

**Anurag Sethi**

USA

**Justin Guinney**

USA

**Samuel Payne**

USA

**Michael Kane**

USA

**Cong Li**

USA

**Faheem Mitha**

USA

**Niina Haiminen**

USA

**W. Evan Johnson**

USA

**Qian Sun**

USA

**Ruilin Liu**

USA

**Lynette Hirschman**

USA

**Iosif Vaisman**

USA

**Radu Herbei**

USA

**Volodymyr Melnykov**

USA

**Donna Karolchik**

USA

**Arindam Roychoudhury**

USA

**Xiaobo Zhou**

USA

**Yang Li**

USA

**Yang Yu**

USA

**David Craig**

USA

**Qi Li**

USA

**Yuqing Mao**

USA

**Zheng Xia**

USA

**Scott Gerber**

USA

**Brian Pratt**

USA

**Laurel Copeland**

USA

**Zhuohui Gan**

USA

**Cathy Lawson**

USA

**Nicole Carnegie**

USA

**Samuel Deutsch**

USA

**Nathan Hillson**

USA

**Rebecca Dickstein**

USA

**Eitan Halper-Stromberg**

USA

**Yakir Reshef**

USA

**Jie Ding**

USA

**Juhee Lee**

USA

**Kenneth Cornetta**

USA

**Libusha Kelly**

USA

**Louis Sherman**

USA

**Jun-Tao Guo**

USA

**Deeptak Verma**

USA

**Xiaobin Zheng**

USA

**Xinan Yang**

USA

**Tong Zhou**

USA

**Vikas Bansal**

USA

**Peter Clote**

USA

**Franz Gruswitz**

USA

**Diana Delibaltov**

USA

**Vignesh Jagadeesh**

USA

**Vesna Memisevic**

USA

**Seongho Kim**

USA

**Jianrong Dong**

USA

**Elena Rivas**

USA

**Joyee Ghosh**

USA

**Jiguang Wang**

USA

**Qin Wang**

USA

**Rasmi Thomas**

USA

**Michael Lawrence**

USA

**Robert Scharpf**

USA

**Dina Schneidman**

USA

**Heiko Enderling**

USA

**Jonas Almeida**

USA

**Scott Edwards**

USA

**Youngik Yang**

USA

**Brian Long**

USA

**Xinping Cui**

USA

**Yen-Tsung Huang**

USA

**Qiong Yang**

USA

**Hetunandan Kamichetty**

USA

**Hua Lou**

USA

**Aiguo Li**

USA

**Zhenyuan Song**

USA

**Luis Tari**

USA

**Dandi Qiao**

USA

**Mohamed Diwan M Abdulhameed**

USA

**Xueping Yu**

USA

**Denis Fourches**

USA

**Robert Kincaid**

USA

**Graham Snyder**

USA

**Gangqing Hu**

USA

**Nikolaus Schultz**

USA

**Caitlin Pepperell**

USA

**Stacey Winham**

USA

**Bernard Kim**

USA

**Tarjei Mikkelsen**

USA

**Matthew Hudson**

USA

**Surya Saha**

USA

**Jean-Francois Rual**

USA

**Ming Wu**

USA

**Zhenqing Ye**

USA

**Ying Ding**

USA

**Benjamin Regner**

USA

**Beisi Xu**

USA

**Zhixing Feng**

USA

**Xuebing Wu**

USA

**Usman Roshan**

USA

**Upadhyayula Surya Raghavender**

USA

**Utkarsh Gaur**

USA

**Vân Anh Huynh-Thu**

USA

**Valerio Bianchi**

USA

**Vasu Punj**

USA

**Vera Van Noort**

USA

**M. Paola Vera-Licona**

USA

**A Mohanapriya**

USA

**Vincent Segura**

USA

**Vitor Sousa**

USA

**Veli Mäkinen**

USA

**Vikas Pejaver**

USA

**Vsevolod Makeev**

USA

**Viktoria Szabo**

USA

**Wolfgang Raffelsberger**

USA

**Wael Ahmed**

USA

**Dirk Walther**

USA

**Qiong Wang**

USA

**Yinan Wan**

USA

**Wei Chen**

USA

**Gang Wu**

USA

**Wibke Busch**

USA

**Wim Verhaegh**

USA

**Wei Jiang**

USA

**Weizhong Li**

USA

**William Dampier**

USA

**William Pearson**

USA

**Mengyun Wu**

USA

**Cen Wu**

USA

**Xiangqin Cui**

Israel

**Rui Xia**

USA

**Xiaoming Wang**

Belgium

**Xiaoqing Yu**

Italy

**Xiaowei Zhou**

USA

**Xiaoxiao Liu**

Belgium

**Xiaxia Yu**

USA

**Xin Wang**

USA

**Xing Chen**

India

**Xinghua Lu**

France

**Xingyu Li**

Switzerland

**Xiting Yan**

Finland

**Xiu Huang**

USA

**Xiaodong Liu**

Russia

**Xueqiu Jian**

Spain

**Jianpeng Xu**

France

**Xujing Wang**

Egypt

**Xiang Wan**

Germany

**Xinan Yang**

USA

**Yanay Ofran**

USA

**Yang Du**

USA

**Yanni Sun**

USA

**Yao Fu**

USA

**Yarden Katz**

Germany

**Yu Fan**

Netherlands

**Yon Hui Sarah Kim**

Hong Kong

**Yin Liu**

USA

**Zhao Yinjun**

USA

**Yoji Nakamura**

USA

**Yasset Perez-Riverol**

China

**Yao Shen**

USA

**Yuan Jiang**

USA

**Yunfei Li**

USA

**Yun Xu**

Canada

**Yupeng Cun**

USA

**Yuping Zhang**

USA

**Yuwen Liu**

USA

**Ioannis Vlachos**

USA

**Yingying Wei**

Hong Kong

**Yoav Broza**

China

**Yate-Ching Yuan**

USA

**Zachary Charlop-Powers**

USA

**Osvaldo Zagordi**

USA

**Wei Zhang**

USA

**Yi Zhao**

USA

**Zhengqing Ouyang**

USA

**Zhen Xie**

USA

**Zheyang Wu**

USA

**Fangqing Zhao**

China

**Zhi Zhou**

China

**Zhizhuo Zhang**

USA

**Zhou Xiaoyuan**

Israel

**Bin Zhu**

Germany

**Ziding Zhang**

USA

**Urs Ziegler**

USA

**Sajjad Karim**

USA

**Shihua Zhang**

USA

